# Supplementary material for: The impact of sleep on eyewitness identifications
Source: R Soc Open Sci. 2019 Dec 4;6(12):170501. doi: 10.1098/rsos.170501 (PMC6936295; doi:10.1098/rsos.170501)
Supplement: Read me file for dataset [file rsos170501supp2.pdf]

The data accompany the paper, Morgan, Tamminen, Seale-Carlisle, & Mickes (under stage 2 review). Examining the impact of sleep on eyewitness identifications. *Royal Society Open Science.*

File name: Morgan_etal_RSOS_ArchivedData.xlsx

The file contains two worksheets “Key” and “Raw Data.”

“Key” contains descriptions of the data in each column of “Raw Data.”

“Raw Data” contains all of the data (N = 4,000)

Participants were randomly assigned to one of four conditions (e.g. AM Control, PM Control, Sleep or Wake). Before the study phase, participants answered pre-screening questions, provided demographic information, answered sleep related questions, and completed the Stanford Sleepiness Scale (SSS). Next, participants were shown a video of mock crime (the study phase) and completed a short distractor task. Before the test phase, participants were asked if they had consumed any caffeine. Participants were then shown a target-absent or target-present lineup (the test phase). They responded that the target was present (and selected someone) or absent and rated their confidence. Participants in the wake condition were then asked if they had napped between phases. All participants completed the SSS, reduced Morningness Eveningness Questionnaire, Epworth Sleepiness Scale, and St Mary's Hospital Sleep Questionnaire. Participants in the sleep condition also answered the sleep related questions a second time. Lastly, participants answered a validation question.

Participants in the AM and PM control conditions completed the study and test phases in one session. Participants in the wake condition completed the study phase any time between 8 a.m.-11 a.m. and the test phase any time between 8 p.m.-11 p.m. the same day. Participants in the sleep condition completed the study phase any time between 8 p.m.-11 p.m. and completed the test phase any time between 8 a.m.-11 a.m. the next morning.
